# Regulatory T cells and immune escape in HCC: understanding the tumor microenvironment and advancing CAR-T cell therapy

**DOI:** 10.3389/fimmu.2024.1431211

**Published:** 2024-07-29

**Authors:** Guangtan Du, Cunmiao Dou, Peng Sun, Shasha Wang, Jia Liu, Leina Ma

**Affiliations:** ^1^ Department of Oncology, The Affiliated Hospital of Qingdao University, Qingdao, China; ^2^ Medical Department of Qingdao University, Qingdao, China; ^3^ Department of Hepatobiliary and Pancreatic Surgery, The Affiliated Hospital of Qingdao University, Qingdao, China; ^4^ Department of Pharmacology, School of Pharmacy, Qingdao University, Qingdao, China; ^5^ Qingdao Cancer Institute, Qingdao, China

**Keywords:** hepatocellular carcinoma (HCC), tumor microenvironment, CAR-T, regulatory T cells(Treg), immunotherapy

## Abstract

Liver cancer, which most commonly manifests as hepatocellular carcinoma (HCC), is the sixth most common cancer in the world. In HCC, the immune system plays a crucial role in the growth and proliferation of tumor cells. HCC achieve immune escape through the tumor microenvironment, which significantly promotes the development of this cancer. Here, this article introduces and summarizes the functions and effects of regulatory T cells (Tregs) in the tumor microenvironment, highlighting how Tregs inhibit and regulate the functions of immune and tumor cells, cytokines, ligands and receptors, etc, thereby promoting tumor immune escape. In addition, it discusses the mechanism of CAR-T therapy for HCC and elaborate on the relationship between CAR-T and Tregs.

## 1 Introduction

HCC is a complex malignancy, with its tumor microenvironment playing a crucial role in promoting tumor growth, invasion, metastasis, and immune escape. This microenvironment is composed of a variety of cellular and molecular components, including tumor cells, cancer-associated fibroblasts (CAFs), vascular cells, and a range of immune cells such as tumor-associated macrophages, bone marrow-derived suppressor cells, Tregs, and liver-specific Kupffer cells. While many of these components contribute to tumor growth and metastasis, certain immune cells, including dendritic cells, regulatory B cells, and tumor-associated neutrophils, exhibit tumor-suppressing effects. Additionally, the tumor microenvironment contains stromal tissue that provides structural support, as well as metabolic products and circulating DNA.

In the immune escape mechanism of HCC, Tregs inhibit the immune cells’ ability to kill HCC through multiple pathways, thereby promoting immune escape of HCC cells. Tregs are closely associated with the poor prognosis of HCC patients. They not only suppress excessive immune responses to prevent impairment of normal functions but also promote the formation of immune tolerance, thus protecting liver cancer cells from systemic attacks. Additionally, studies have shown that an excess of Tregs in liver cancer patients is linked to poor treatment outcomes because Tregs limit the effectiveness of immunotherapy and weaken the efficacy of immune checkpoint inhibitors.

Unlike many other tumors, liver cancer often develops from chronic liver disease and possesses a unique dual blood supply from the hepatic artery and portal vein. This dual supply facilitates the invasion and metastasis of liver cancer. Additionally, the liver functions as an immune regulatory center, aggregating various types of immune cells and creating distinct immune characteristics. These properties significantly impact the physiological state of the liver and cause Tregs in liver cancer to exhibit different expression patterns compared to other tumors. For instance, in liver cancer, Tregs frequently express high levels of Programmed Death-Ligand 1 (PD-L1) and Cytotoxic T-lymphocyte-associated protein 4 (CTLA4). These unique features significantly increase the complexity and challenge of researching and treating liver cancer.

## 2 Regulatory T cells and hepatocellular carcinoma

The immune microenvironment in HCC is predominantly influenced by Tregs, which play a crucial role in immune suppression and regulation, significantly affecting the progression of liver cancer. This relationship prompts an in-depth exploration of the interaction between regulatory T cells and liver cancer.

### 2.1 Regulatory T cells

Regulatory T cells, a subpopulation of T lymphocytes, possess immunosuppressive capabilities. They regulate immune responses by directly inhibiting activated immune cells to prevent an overactive immune response. Additionally, they achieve immunosuppression by releasing cytokines and other substances that inhibit the activation of immune cells.

Treg research involves multiple fields, including the pathogenesis and treatment of various autoimmune diseases, cancer and allergy, as well as transplantation research and tissue engineering. Here, it focuses on regulatory T cells in hepatocellular carcinoma. It is found following the development of liver cancer, a significant increase in Tregs is observed in the tumor microenvironment of the cancerous part, which affect the development and treatment outcomes of hepatocellular carcinoma through various mechanisms. This raises the question: what is the origin of these abundant Tregs in liver cancer? After the onset of liver cancer, dendritic cells in the lesion site recognize and present antigens, activating a large number of T cell responses. Studies have shown that transforming growth factor-beta (TGF-β) has been shown to promote the plasticity of Th17 cells in a dose-dependent manner facilitating their conversion into Treg cells (1). Additionally, it has been demonstrated that when activated T cells are co-cultured with exosomes derived from HCC, there is an induced conversion of CD4^+^ T cells into Tregs (2). Regulatory T cells express high levels of chemokine receptors such as Characteristic Chemokine Receptor 4 (CCR4), Characteristic Chemokine Receptor 8 (CCR8), Characteristic Chemokine Receptor 10 (CCR10), and C-X-C Motif Chemokine Receptor 3 (CXCR3). In liver cancer, chemokines such as C-C Motif Chemokine Ligand 17 (CCL17), C-C Motif Chemokine Ligand 22 (CCL22), C-C Motif Chemokine Ligand 28 (CCL28), and C-X-C Motif Chemokine Ligand 9 (CXCL9) are produced, which bind to these receptors, and consequently recruit a substantial number of Tregs to the liver cancer site (3). Furthermore, interleukin-10 secreted by tumor-associated macrophages (TAMs) also activate regulatory T cells (4).

### 2.2 Regulatory T cells and the immune microenvironment of hepatocellular carcinoma

Previous studies have shown that regulatory T cells play an inhibitory role in hepatocellular carcinoma. In this section, it mainly discusses how Treg cells exert their inhibitory effects in the tumor microenvironment of hepatocellular carcinoma.

#### 2.2.1 Immune cells

Immune cells are vital components of the tumor microenvironment (TME), with different immune cells playing distinct roles in the tumor microenvironment. Some immune cells infiltrate the TME to exert cytotoxic effects on HCC, including T cells, Natural killer cell (NK cell), B cells, Dendritic cells (DCs) and Neutrophils, etc.

As shown in the **Supplementary Table**, in the tumor microenvironment of HCC, infiltrating T cells include CD8^+^ cytotoxic T lymphocytes (CTLs), natural killer T (NKT) cells, regulatory T cells, and helper T cells. CD8^+^ CTLs target tumor cells by recognizing specific antigens and secreting cytotoxic substances, while NKT cells link the innate and adaptive immune systems, with type I NKT cells exerting anti-tumor effects and type II NKT cells promoting tumor growth through immunosuppressive actions (5). Different levels of CD40 expression alter T cell responses through different mechanisms: first, CD40 signaling affects the process of antigen processing and presentation, and second, different levels of CD40 produce different levels of IL-12 and IL-10 (6).

NK cells activate their cytotoxicity by recognizing stress molecules on the surface of tumor cells, subsequently destroying the tumor cell membrane and inducing apoptosis through the release of perforins and granzymes. For B cells, B cells primarily include activated B cells, depleted B cells, and plasma cells, with plasma cells producing and secreting antibodies. Additionally, the heightened glucose and oxygen consumption by activated B cells and plasma cells depletes the tumor microenvironment of essential nutrients and energy, thereby impairing the effector function of immune cells (7, 8).

In HCC, the two main subtypes of dendritic cells (DCs) are conventional DCs, which maintain self-tolerance and induce specific immune responses by presenting liver-acquired antigens to T cells (9, 10), and plasmacytoid DCs, which circulate in the bloodstream and is either tolerogenic when immature or immunogenic and cytokine-secreting when mature (11, 12). CD40 activation on dendritic cells enhances cytokine and chemokine production, induces costimulatory molecules, and facilitates antigen cross-presentation, thus improving DC-T cell interactions and promoting anti-tumor effects. As the most abundant type of granulocytes in innate immunity, neutrophils are crucial immune cells active during liver inflammation and injury. Regarding neutrophils, neutrophils are divided into type 1 and type 2 subsets. Type 1 neutrophils exert a pro-inflammatory effect by producing interleukin 12 (IL-12) and C-C Motif Chemokine Ligand 3 (CCL3), while Type 2 neutrophils are immunosuppressive secreting IL-10 and C-C Motif Chemokine Ligand 12 (CCL2) (13). Additionally, myeloid-derived suppressor cells (MDSCs) inhibit T cell proliferation and activation by depleting essential amino acids through elevated arginase activity (14).

Tumor-associated macrophages (TAMs) exhibit two distinct phenotypes: the M1 phenotype, which expresses TGF-α, IL-12, and other factors that exert pro-inflammatory and anti-tumor effects, and the M2 phenotype, which expresses IL-10, TGF-β, and other factors that exert anti-inflammatory and pro-tumor effects (15). CD40 signaling also shifts macrophages from the M2 to the more tumoricidal M1 phenotype (16). In the liver, Kupffer cells, which are resident macrophages, play a key role in the formation and development of HCC by mediating inflammatory responses, fibrosis, and immune cell recruitment, thereby affecting the balance between pro-fibrotic and anti-fibrotic processes. Additionally, liver cell death induces the formation of apoptotic bodies, which are engulfed by Kupffer cells, leading to a pro-fibrotic response that promotes the development of HCC (17).

Resting Treg cells express and release TGF-β on their surface, which then bind to TGF-β receptors on the surface of resting NK cells. This interaction inhibits the proliferation and cytotoxic functions of NK cells (18). Naive B cells contribute to enhancing immune tolerance and facilitating the differentiation of Treg cells (19). Additionally, B cells support their own differentiation into IgA-producing plasma cells by stimulating the proliferation of Treg cells (20). In the case of macrophages, they recruit mature Treg cells into HCC and promote the transformation of naive T cells into Treg cells by secreting cytokines such as CCL22, CCL20, TGF-β, and IL-10 (21). Pro-tumor neutrophils recruit Treg and anti-inflammatory M2 macrophages and help further inhibit the function of cytotoxic T cells (22). In addition, LPS-stimulated Treg has been found to induce IL-10 production in neutrophils in a cellular contact-dependent manner. Additionally, MDSCs stimulate the differentiation and development of Tregs during tumor progression and inhibit NK and DC cell functions through TGF-β to promote tumor immune escape (23).

#### 2.2.2 Stromal cells

In HCC, in addition to immune cells, stromal cells are present, forming part of the extracellular matrix. These stromal cells include cancer-associated fibroblasts (CAFs) and hepatic stellate cells (HSCs). CAFs participate in aerobic glycolysis and secrete lactate, which promote the metabolism of cancer cells (24). Increased secretion of metabolites by CAFs along with reorganization of Extracellular Matrix (ECM) proteins, enhances collagen deposition, leading to ECM stiffening (25). This stiffened extracellular matrix not only enhances tumor cell adhesion but also disrupts intercellular contact, preventing contact inhibition between cells, thereby promoting the growth and survival of cancer cells. Additionally, the stiffened extracellular matrix impedes drug uptake into the tumor area, enhancing the survival of tumor cells, reducing cancer cell death and the release of cancer cell antigens, and weakening immune therapy for cancer.

During HCC development, HSCs are activated by fibrosis in liver stromal spaces, transitioning from a quiescent state to an activated myofibroblast phenotype with the ability to release collagen and ECM remodeling factors. In addition, activated HSCs secrete angiogenesis growth factors to form new vasculature in the TME (26, 27). These activities of activated HSCs establish connections with the circulatory system, providing tumors with the nutrients needed for growth and metastasis.

**Figure 1:**
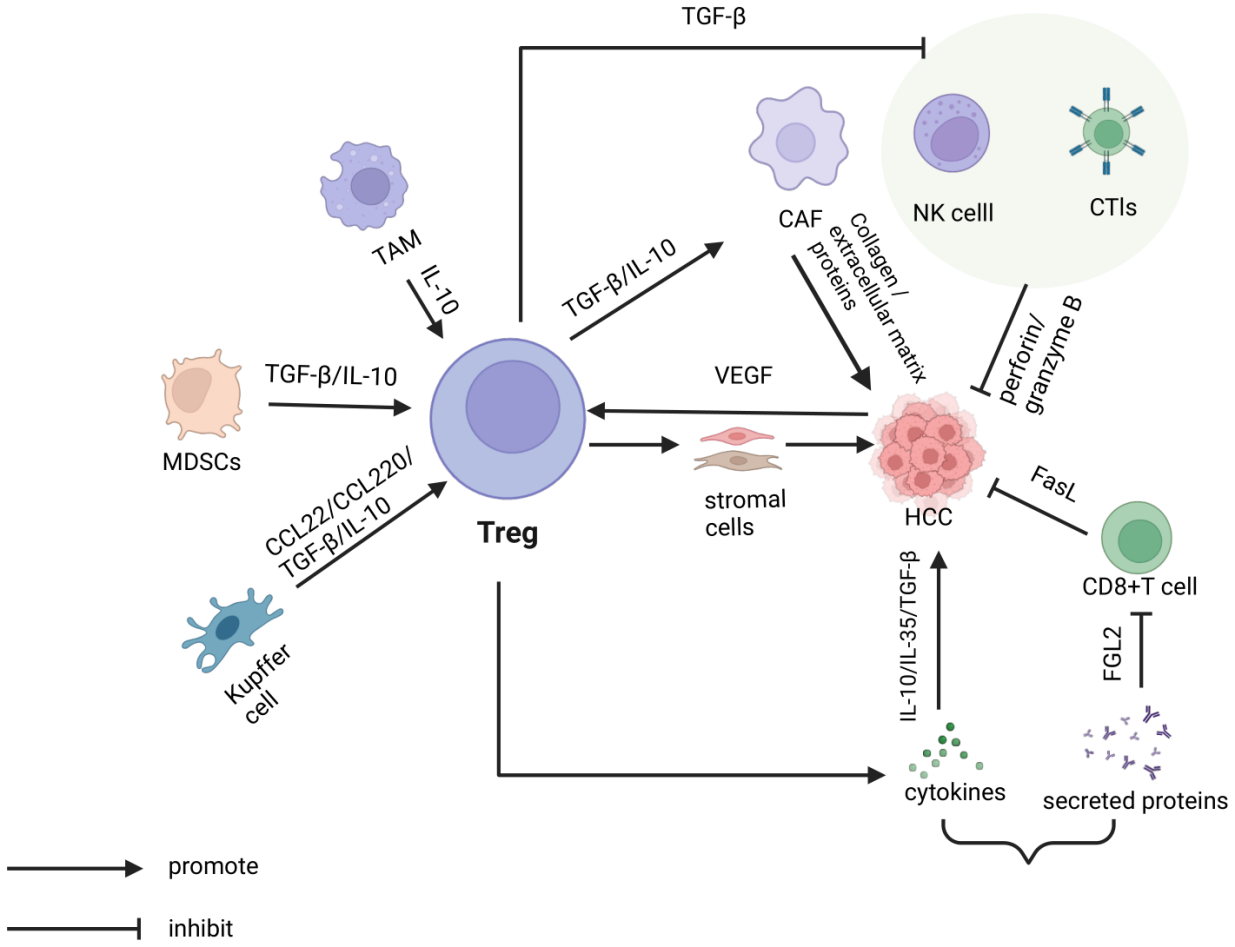
In the tumor microenvironment of hepatocellular carcinoma, various cells indirectly promote the escape of tumor cells by regulating T cells. This includes tumor-associated macrophages (TAMs), myeloid-derived suppressor cells (MDSCs), and Kupffer cells, which release cytokines such as TGF-β and IL-10, as well as chemokines like CCL22. Additionally, Tregs directly promote tumor cell escape by secreting immunosuppressive cytokines, including TGF-β, IL-10, and IL-35. On one hand, tregs secrete TGF-β and IL-10 to stimulate cancer-associated fibroblasts (CAFs). These CAFs then produce collagen and extracellular matrix components, which facilitate tumor cell escape. On the other hand, tregs inhibit the cytotoxic activity of natural killer (NK) cells, cytotoxic T lymphocytes (CTLs), and CD8^+^T cells against tumor cells through the secretion of TGF-β and proteins such as FGL2.

Tregs interact with these stromal cells, influencing the progression of HCC. Tregs promote the activation and transformation of CAFs by secreting inhibitory cytokines IL-10 and TGF-β. This causes CAFs to produce more collagen and extracellular matrix proteins, thereby facilitating the invasion and metastasis of tumor cells (28). Regarding HSCs, studies have shown that activated HSCs significantly upregulate the expression of Treg cells, thereby promoting the growth of HCC in the spleen, bone marrow, and other tissues (29) (**Figure 1**).

#### 2.2.3 Cytokines

In previous studies, it discussed that Treg cells exert inhibitory effects either through direct cell-to-cell contact or by releasing cytokines that act on other cells. This article focuses primarily on the role of cytokines within the immune microenvironment.

##### 2.2.3.1 Enzymes

In the immune microenvironment, enzymes secreted by various cells influence the progression of HCC through distinct pathways. In the tumor microenvironment, enzymes like intracellular glucose-6-phosphate dehydrogenase (G6PD) are involved in various metabolic pathways, such as the pentose phosphate pathway (PPP). NADPH primarily functions in defending against reactive oxygen species and in lipid biosynthesis, while ribose, produced as a result, provides a saccharide phosphate backbone essential for nucleotide synthesis (30). The defensive role of NADPH against reactive oxygen species enhances the ability of cancer cells to withstand oxidative stress (31), thereby facilitating their evasion from immune system clearance. Ribose, as a metabolite of glucose, provide the energy needed for the growth and metabolism of tumor cells. In addition, ribose is also an essential substrate for DNA synthesis, which promote the proliferation and growth of tumor cells (32). In normal tissues, the tumor suppressor gene p53 binds to G6PD, preventing the formation and activation of G6PD dimers, and thus inhibiting tumor development. However, in cancer, TAp73, a member of the p53 family, is often overexpressed, leading to the induction of G6PD expression which promotes tumor growth (33, 34).

Tumor cells secrete matrix metalloproteinases (MMPs), enzymes that degrade the extracellular matrix, enabling the tumor cells to penetrate the basement membrane and invade surrounding tissues and blood vessels, thereby promoting liver cancer metastasis (35).

Ecto-nucleotidases CD39 and CD73 are expressed on the surface of regulatory T cells. Under normal conditions, ATP is strictly retained within the cell. However, research has shown that following cellular damage, intracellular ATP is released into the extracellular space through vesicle exocytosis and membrane transporters. CD39 on the surface of Treg cells then metabolizes ATP into ADP and AMP, and CD73 subsequently converts AMP into adenosine. The adenosine produced binds to adenosine receptors, inhibiting the function of effector T cells (36).

##### 2.2.3.2 Vascular endothelial growth factor

In the TME, cancer-associated fibroblasts (CAFs) secrete vascular endothelial growth factor (VEGF) to stimulate the formation of new blood vessels, enabling tumor cells to metastasize through these newly formed blood vessels. Additionally, VEGF inhibits the differentiation of dendritic cells into their mature cells, thus suppressing the antigen-presenting function of mature dendritic cells and the cytotoxic effects of effector T cells, favoring immune evasion by tumor cells (37).

Tumor cells, inflammatory cells, and damaged tissues all produce VEGF (vascular endothelial growth factor). Elevated levels of VEGF stimulate the proliferation and accumulation of Tregs. Tumor-derived VEGF serve as a chemotactic agent, attracting Tregs to the tumor microenvironment (38). Furthermore, VEGF enhance the immunosuppressive function of Tregs by regulating signaling pathways, such as STAT3, to promote Treg differentiation and stability (39).

##### 2.2.3.3 Transforming growth factor-β

In the tumor microenvironment, various cells secrete transforming growth factor-beta (TGF-β). For example, TGF-β secreted by cancer-associated fibroblasts (CAFs), induces hepatocellular carcinoma cells to undergo epithelial-mesenchymal transition (EMT), where the cells transition from an epithelial to a mesenchymal phenotype. This EMT process causes liver cancer cells to lose their adhesive ability and polarity, increasing their migration and invasion capabilities, thereby promoting immune evasion in liver cancer (40).

Kupffer cells, a type of macrophage, exhibit both M1 and M2 phenotypes. As liver cancer progresses, Kupffer cells transition from an intermediate phenotype to the M1 phenotype, characterized by an anti-inflammatory profile. When exposed to factors like IL-3 and IL-4, M1 cells secrete TGF-β. With the increasing levels of TGF-β in the TME, Kupffer cells gradually transition from an anti-inflammatory to an immune-suppressive phenotype, promoting immune evasion (17, 41).

Myeloid-derived suppressor cells (MDSCs) release TGF-β to inhibit the activity and function of natural killer (NK) cells, leading to immune evasion in tumors. For instance, TGF-β inhibits the secretion of IFN-λ by NK cells and reduces the expression of activation receptors on their surfaces, thereby impairing the recognition and elimination of target cells. TGF-β also act back on MDSCs, inducing them to secrete inhibitory molecules such as IDO, further suppressing NK cell function (42).

In the immune microenvironment, various cells secrete transforming growth factor-beta (TGF-β). For instance, regulatory T cells secrete TGF-β, which diminish the cytotoxic functions of natural killer (NK) cells and cytotoxic T lymphocytes (CTLs). This process induces NK cells to transform into type 1 innate lymphoid cells within the tumor microenvironment, reducing their ability to suppress tumor growth and metastasis (43).

##### 2.2.3.4 Interferon-γ

In HCC, IFN-γ is primarily produced by activated T cells and NK cells, playing a crucial role in immune regulation. Research has shown that in HCC, IFN-γ synergize with IL-1β to enhance the expression of IRF-1, which subsequently leads to an increase in PD-L1 expression (44). Moreover, IFN-γ induce a state in tumor-derived Tregs where they exhibit a reduction in their suppressive activity (45). Moreover, IFN-γ induce a state in tumor-derived Tregs where they exhibit a reduction in their suppressive activity.

##### 2.2.3.5 Interleukin 10 and Interleukin 35

Regulatory T cells secrete inhibitory factors for immune suppression, such as IL-10 and IL-35. IL-10 has broad immunosuppressive and anti-inflammatory effects. Its main mechanisms include regulating the activity of inflammatory cells. For example, IL-10 upregulate mature protein-1 induced by B lymphocytes to inhibit CD28 tyrosine phosphorylation and induce exhaustion of CD8^+^ T cells (46). It also reduces the production of inflammatory mediators, lowers the intensity of inflammatory reactions, inhibits antigen presentation and co-stimulatory signal provision, and promotes the formation of immune tolerance (47). IL-10 has a potent immunosuppressive effect on APC and effector T cells, and convert DCs to tolerant DCs. In addition, local production of IL-10 lead to the exclusion of APC from the tumor mass (48). IL-35 has extensive immunosuppressive effects and is considered a negative regulatory factor. It suppress the immune response through pathways such as inhibiting cell proliferation, reducing the production of inflammatory mediators, promoting the development and function of Treg cells, assisting immune escape. For instance, IL-35 induces T cells to express inhibitory receptors like PD-1, TIM3, LAG3, facilitating T cell exhaustion and contributing to immune escape (49). IL-35 has also been found to inhibit the production of IFN-γ, thus suppressing the development of inflammation through this pathway (50).

#### 2.2.4 Immune-related proteins

##### 2.2.4.1 Leukocyte function-associated antigen-1

One integral protein expressed on the surface of regulating T cells is Leukocyte Function-Associated Antigen-1 (LFA-1). Under normal conditions, LFA-1 on regulatory T cells facilitates interaction with dendritic cells, supporting the initiation and regulation of immune responses, supporting T cells in recognizing and responding to tumor cells. However, regulatory T cells, upon stimulation of antigen receptors, form aggregates with DCs through LFA-1, inhibiting the co-stimulatory process between DCs and effector T cells (51).

##### 2.2.4.2 V-domain Ig suppressor of T-cell activation

VISTA, a B7 family member, crucially maintains T cell quiescence and regulates bone marrow cell populations. In HCC, higher VISTA levels correlate with CD8+ tumor-infiltrating lymphocytes (TILs) (52). VISTA is more expressed in tumor-infiltrating Tregs compared to those in peripheral lymph nodes, indicating its role in suppressing tumor-specific immunity within the tumor microenvironment (TME) (53). VISTA-Ig fusion protein has been shown to inhibit T cell activation by blocking proliferation and cytokine production (54). Moreover, VISTA inhibits iTreg transformation to Th1 and Th17 in inflammatory environments, while promoting iTreg differentiation under TGF-β induction *in vitro* (55). On the other hand, VISTA-Ig promotes the differentiation of iTregs in response to *in vitro* TGF-β induction (53).

##### 2.2.4.3 Cytotoxic T-lymphocyte associated protein 4

Regulatory T cells assist in immune suppression by secreting various immune-related proteins. For example, CTLA-4, expressed on Tregs, negatively regulates T cell activation by binding to CD80/CD86 on APCs, thus blocking the stimulatory effects of CD28 binding to these molecules. Both CD28 and CTLA-4 have affinities for CD80/CD86, but they have different effects on co-stimulatory signal transduction. Binding of CD28 to CD80/CD86 provides a second signal that enhances T cell activation, proliferation, and function. In contrast, when CTLA-4 binds to CD80/CD86, it competitively antagonizes the signaling of CD28, inhibiting its signal transduction. Through this mechanism, Tregs suppress T cell activation, contributing to immune escape of cancer cells (56).

#### 2.2.5 Chemokines

Chemokines are a class of small cytokines knowns for their similar structures, functions, and chemotactic properties. They directly act on tumor cells and non-immune cells such as vascular endothelial cells, regulating the proliferation, invasion, and metastasis of tumor cells, thereby promoting the progression of cancer in the tumor microenvironment (57).

CCR8^+^Treg cells are recruited to tumors by C-C Motif Chemokine Ligand 1 (CCL1), which promotes immune escape. CCL1 also induces STAT3-dependent upregulation of FOXP3, CD39, and IL-10, thus enhancing the immunosuppressive activity of Tregs (58). Another way chemical factors promote tumor development is by mediating the entry of Tregs into the tumor microenvironment. Treg cells express CCR4 and are recruited to the tumor microenvironment by CCL22 induction. In addition to the CCL22-CCR4 pathway, Treg cells express CCR10 and respond to migration cues like CCL28 in hypoxic areas of the tumor microenvironment (59, 60).

Bone marrow, a common site for tumor metastasis, hosts numerous Treg cells with a memory phenotype and functional C-X-C Motif Chemokine Ligand 4 (CXCR4) expression. Treg cells move from bone marrow to peripheral blood through the action of granulocyte colony-stimulating factor (G-CSF), which promotes the degradation of C-X-C Motif Chemokine Ligand 12 (CXCL12) in the bone marrow. The abundance of Treg cells in the bone marrow may provide an immune “shield,” favoring tumor metastasis to this site. Treg cells producing C-X-C Motif Chemokine Ligand 8 (CXCL8) and IL-17 not only suppress T cells but also promote inflammation within the cancer microenvironment.

#### 2.2.6 Metabolites

Substances produced by the metabolism of components in the tumor microenvironment of liver cancer also impact the formation and development of tumor cells. Metabolites in the microenvironment interact with various cells to exert their effects. In hepatocellular carcinoma, the Warburg effect leads tumor cells to utilize glucose via aerobic glycolysis—even under oxygen-rich conditions—to produce ATP, concurrently increasing lactate production and lowering pH in the tumor microenvironment (61). High lactate production in the tumor microenvironment increases FOXP3 concentration. Under normal circumstances, FOXP3 promotes the growth of Tregs and limits the differentiation of Tregs into other immune cells, maintaining the stability and function of Treg cells (62). The metabolic processes of tumor cells transform the tumor microenvironment into a nutrient-restricted, hypoxic, and lactate-rich environment. In tumors, Tregs convert pyruvate into acetyl-CoA via mitochondria, utilizing the tricarboxylic acid cycle to sustain survival (63). Furthermore, Tregs have a unique fatty acid metabolism mechanism where lipid oxidation in Tregs reduces their glucose requirements and mitigates the cytotoxic effects induced by fatty acids. Even under nutrient-restricted conditions, Tregs utilize fatty acids for proliferation and to perform their immunosuppressive functions (64).

### 2.3 Exosomes

Exosomes are small vesicles containing a variety of biomolecules that mediate intercellular communication, thus regulating the microenvironment and immune system interactions. Research has shown that exosomes not only transmit downstream signals to target cells but also transfer genetic material to downstream cells, thus facilitating intercellular communication (65).

Apart from their communicative role, exosomes participate in various processes in hepatocellular carcinoma, including tumor survival, growth, angiogenesis, and invasion. Exosomes transfer biological molecules such as miRNAs, lncRNAs, and circRNAs, which are primarily involved in regulating interactions between HCC cells and endothelial cells, thereby stimulating angiogenesis to supply oxygen and nutrients to the tumor. Exosomes from cancer-associated fibroblasts (CAFs) modulate liver cancer cell phenotypes by transferring miRNAs, proteins, and miRNA-mediated RNA oxidases, thus activating signaling pathways like Wnt/β-catenin and promoting epithelial-mesenchymal transition (EMT), which enhances migration and invasion capabilities (66). Tumor-derived exosomes promote M2 polarization and increase PD1 expression on T cells, while concurrently inhibiting cytokines that activate M1 macrophages and their phagocytic activity (67, 68). In addition to tumor sources, exosomes secreted by macrophages express specific CD11b/CD18 proteins to promote MMP-9 expression, thereby promoting HCC migration (69).Furthermore, exosomes alter the stromal environment surrounding the tumor by regulating the synthesis and degradation of matrix components and matrix adhesion factors, thereby creating a suitable tumor microenvironment and enhancing the metastatic ability of hepatocellular carcinoma (70). Certain molecules within exosomes regulate the function and immune response of immune cells. They suppress the activity of immune cells, inhibit the cytotoxic effects of natural killer cells and T cells, thereby helping liver cancer cells evade immune attacks and metastasis (71). In the tumor microenvironment, exosomes package circular RNAs that suppress immunity in HCC, for instance, by increasing CD39 expression in macrophages, which contributes to resistance against anti-PD-1 therapy (72).

## 3 Research progress of CAR-T cell therapy for hepatocellular carcinoma

**Figure 2:**
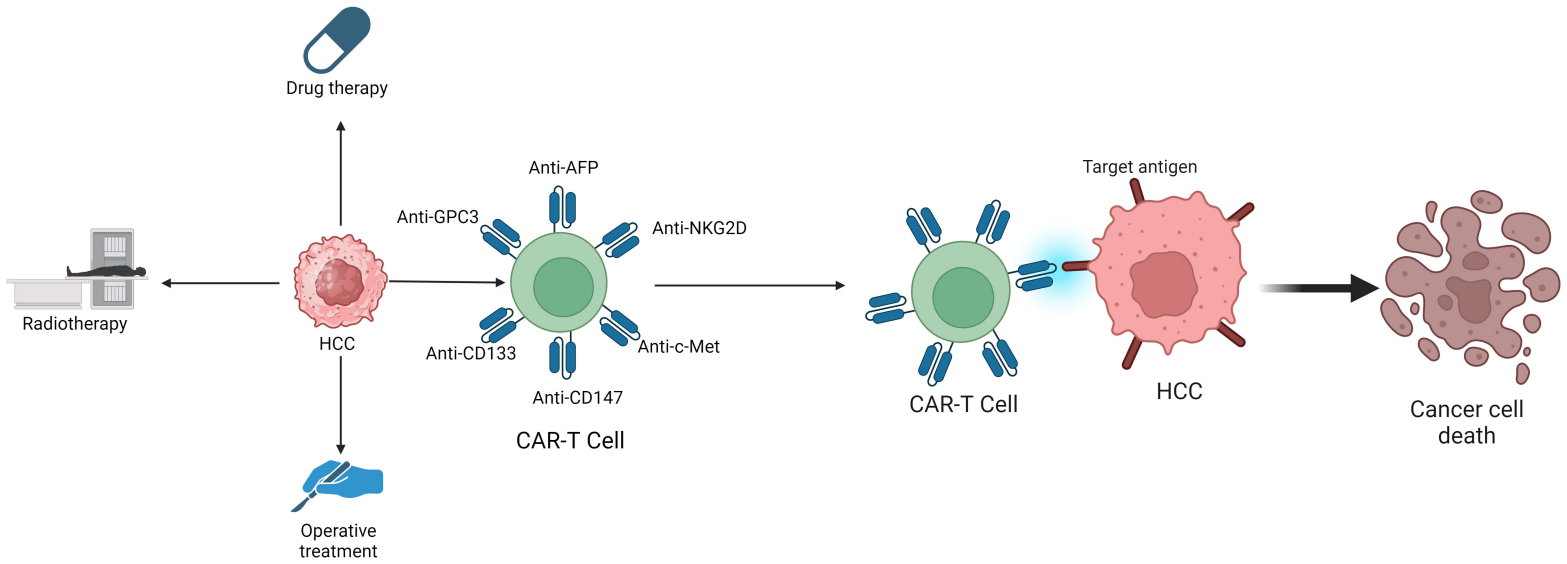
The treatment methods for HCC include Drug therapy, Radiotherapy, Operative treatment and CAR-T Cell therapy.

Common treatments for hepatocellular carcinoma include drug therapy, radiation therapy, and surgical interventions, such as donafenib, apatinib, durvazumab, and sorafenib. However, these traditional therapies often result in low cure rates and poor prognosis, whether used alone or in combination (73). This underscores the urgent need for innovative treatments. Consequently, to improve treatment outcomes in hepatocellular carcinoma, researchers have developed a novel immunological approach—CAR-T therapy (**Figure 2**). CAR-T therapy is chimeric antigen receptor T-cell immunotherapy, which is also known as Chimeric Antigen Receptor T-Cell Immunotherapy. CAR-T therapy, a precise form of targeted tumor treatment, has recently made significant advancements in liver cancer treatment. A CAR-T cell is a special type of cell in T cells. CAR consists of three parts: an extracellular antigen recognition domain with or without hinge/spacer domains, a transmembrane domain, and an intracellular signal domain. The hinge/spacer ensures the accessibility of CAR-T cell epitopes, making CAR expression very stable. The transmembrane domain affect the expression of CAR on the cell surface and signal transduction in T cells. The intracellular signaling domain comprises two parts: the costimulatory and signal transduction domains. The costimulatory domain typically includes either the CD28 receptor family (CD28,ICOS) or the tumor necrosis factor receptor family (4–1BB,OX40,CD27). The role of the costimulatory domain is to achieve synergistic stimulation of the dual activation of molecular and intracellular signals, and improve T cell action and anti-tumor ability. However, CD28 and 4–1BB have different effects, and CD28 cause CAR-T cells to be metabolized by glycolysis, which promote the differentiation of CAR-T cells into effector T cells. 4–1BB promote mitochondrial production, enhance respiration and fatty acid oxidation, and CAR-T cells will differentiate into central memory T cells after exposure to antigen stimulation (74–76). In addition to this, CD28 exhibits faster and stronger signaling activity, while 4–1BB is comparatively slower and gentler, and studies have found that 4–1BBnbetter prolong T cell lifespan and maintain its anti-cancer effects (77). Based on the number of costimulatory molecules, CAR proteins are classified into three generations (78). Each of the three generations of CAR proteins plays a crucial role in treating hepatocellular carcinoma. A key advantage of CAR-T therapy in treating hepatocellular carcinoma is that its efficacy does not rely on antigen presentation by major histocompatibility complex (MHC) molecules, thus circumventing MHC-related restrictions and addressing tumor immune escape issues due to MHC downregulation (79). The treatment of hepatocellular carcinoma through CAR-T start with the types of CAR-T, treatment targets for hepatocellular carcinoma, and substances secreted by CAR-T.

### 3.1 Strategies for CAR-T cells therapy for hepatocellular carcinoma

There are many types of CAR-T cells, among which GPC3 CAR-T cells, CD133 CAR-T cells, c-Met CAR-T cells, NKG2D CAR-T cells, and other CAR-T cells effectively treat hepatocellular carcinoma (80).

#### 3.1.1 Glypican-3 CAR-T cell

Glypican-3 (GPC3) is a carcinoembryonic heparan sulfate proteoglycan that attaches to cell membranes via glycophosphatidylinositol anchors (81). It is highly expressed in hepatocellular carcinoma but absent in normal tissues, potentially playing a critical role in regulating cell division and growth. Numerous studies have demonstrated that higher GPC3 expression in hepatocellular carcinoma correlates with poorer prognosis. This suggests GPC3’s potential as a prognostic marker for hepatocellular carcinoma. Notably, GPC3 is highly expressed in hepatocellular carcinoma and squamous non-small cell lung cancer but is expressed at low levels in other cancers. This indicates that GPC3 serve as an important target for the treatment of hepatocellular carcinoma. Therefore, GPC3 CAR-T cells have emerged (82). GPC3 CAR-T cells secrete IL15 and IL21, enhancing the proliferation of poorly differentiated GPC3-CAR T cells, maintaining TCF-1 expression, and increasing both the persistence and anti-tumor activity of these cells. This greatly enhances the treatment of hepatocellular carcinoma by GPC3 CAR-T cells (83). In addition, Lin28 is also overexpressed in hepatocellular carcinoma, and regulating Lin28B enhance the anti-tumor activity of GPC3 CAR-T cells, enabling GPC3 CAR-T cells to better treat hepatocellular carcinoma (84). Researchers have found that generating GPC3-specific CAR-T cells through simultaneous electroporation of plasmid DNA encoding the piggyBac (PB) transposon and overactive piggyBac transposase, rather than using common viral vectors, results in cells with enhanced proliferation and cytokine secretion (85). This discovery has propelled further development of GPC3 CAR-T cell therapy for hepatocellular carcinoma. However, GPC3 CAR-T cell therapy for hepatocellular carcinoma remains in its early stages, with its methods and effects yet to meet clinical needs. Therefore, a large amount of research is still needed to make this treatment method better treat hepatocellular carcinoma.

#### 3.1.2 CD133 CAR-T cell

CD133, which is highly expressed in hepatocellular carcinoma, presents an attractive therapeutic target (86). Studies indicate that CD133 CAR-T cells exhibit effective anti-tumor activity and manageable safety profiles in treating advanced hepatocellular carcinoma. In fact, CD133 is also highly expressed in endothelial progenitor cells of tumors. CD133 promotes angiogenesis in hepatocellular carcinoma; thus, combining CD133 CAR-T cells with anti-angiogenic drugs effectively inhibit new blood vessel formation in tumors. Unlike GPC3 CAR-T cells, CD133 CAR-T cells not only block the nutritional supply to tumor cells but also are used in combination with other drugs to treat hepatocellular carcinoma (87). It should be noted that CD133 CAR-T treatment for hepatocellular carcinoma in patients with biliary obstruction requires more caution (86).

#### 3.1.3 c-Met CAR-T cell

c-Met, a hepatocyte growth factor receptor (also known as HGFR) and a cell surface protein tyrosine kinase, is overexpressed in hepatocellular carcinoma. It plays a crucial role in tumor cell proliferation, invasion, and apoptosis. Overexpression of c-Met is an indicator of increased tumor invasiveness and poor prognosis (88), and because of antigen escape effect and tumor microenvironment and other factors, ordinary CAR-T cells have poor therapeutic effect on hepatocellular carcinoma (89). Therefore, c-Met is an important target for immunotherapy of hepatocellular carcinoma. c-Met CAR-T cells bind to c-Met targets in hepatocellular carcinoma and specifically kill tumor cells in an antigen-dependent manner (88). However, the immunosuppressive tumor microenvironment of hepatocellular carcinoma induce the expression of PD-1 on CAR-T cells, and make PD-1 combine with PD-L1 on tumor cells to inhibit the role of CAR-T cells, so that tumor cells avoid immune surveillance (90, 91). Consequently, constructing CAR-T cells that target both c-Met and PD-1 could enhance therapeutic effects in hepatocellular carcinoma. c-Met/PD-1 CAR-T cells block the interaction between PD-1 and PD-L1, displaying lower levels of inhibitory receptors, reduced differentiation, enhanced anti-tumor activity, and extended survival compared to conventional CAR-T cells (92). Therefore, c-Met/PD-1 CAR-T cells have broad application prospects for better treatment of hepatocellular carcinoma. In addition, various c-Met CAR-T cells such as c-Met/PD-L1 CAR-T cells are being studied to better treat hepatocellular carcinoma.

#### 3.1.4 NKG2D CAR-T cell

NKG2D is a type II transmembrane C-type lectin-like protein receptor expressed on natural killer cells, CD8+ T cells, and some autoreactive CD4+ T cells (93, 94). NKG2DL, the ligand for NKG2D, is not expressed in normal cells but is highly expressed in tumor cells, with expression further inducible by radiotherapy and chemotherapy (95). Therefore, NKG2DL is an important target for the treatment of hepatocellular carcinoma (96). Combining NKG2D CAR-T cells with NKG2DL targets in hepatocellular carcinoma eradicate tumor neovascularization, ameliorate the tumor microenvironment, and augment immunotherapy efficacy (97). Moreover, NKG2D CAR-T cells target multiple ligands expressed in hepatocellular carcinoma to cope with tumor immune escape caused by tumor heterogeneity. Therefore, NKG2D CAR-T cells have the possibility of radical treatment of hepatocellular carcinoma (98). In addition, NKG2D CAR-T cells have good effects and broad application prospects in the treatment of multiple myeloma, glioblastoma, osteosarcoma and other tumors (96).

#### 3.1.5 CD147 CAR-T cell

CD147, a glycoprotein, acts as a regulator of matrix metalloproteinases (MMPs). Numerous studies have shown that CD147 regulate cell proliferation, drug resistance, and cell matrix adhesion characteristics through cell matrix and cell interactions. CD147 is overexpressed in cancer cells and contributes to angiogenesis by regulating vascular endothelial growth factor production in tumor and stromal cells, thus promoting tumor progression. Therefore, CD147 has become a potential therapeutic target for treating hepatocellular carcinoma (99). However, CD147 is not only expressed in cancer cells, but also in small amounts in other tissues, which may result in CD147 CAR-T cells not accurately reaching the CD147 target of hepatocellular carcinoma and may be toxic to normal cells. To minimize the toxicity of CD147 CAR-T cells, researchers have employed the Tet-On 3G system. Tet-On 3G is the third-generation tetracycline induced gene expression system, which use Doxycycline to turn on or off gene expression reversibly. This system allows for the controlled induction of Tet-CD147 CAR-T cells at specific times and locations, optimizing the treatment of hepatocellular carcinoma. Furthermore, in the presence of Doxycycline, Tet-CD147 CAR-T cell activity is significantly enhanced in hepatocellular carcinoma, potentially improving treatment outcomes (100).

#### 3.1.6 Alpha fetoprotein CAR-T cell

Alpha fetoprotein (AFP), a glycoprotein of the albumin family, is primarily synthesized by fetal liver cells and the yolk sac. The content of AFP in normal adult serum is extremely low. Research indicates that AFP promotes cell proliferation and inhibits apoptosis; thus, elevated levels are commonly associated with tumors. Alpha fetoprotein (AFP) is the most commonly used biomarker in hepatocellular carcinoma, often used for detection, diagnosis, and prognosis (101). Therefore, AFP is a good target for the treatment of hepatocellular carcinoma. Traditional CAR-T cell therapy for hepatocellular carcinoma cause serious side effects, including cytokine release syndrome (CRS), and the AFP peptide-MHC complex is less expressed on the surface of tumor cells. AFP CAR-T cells effectively kill tumor cells without inducing cytokine release syndrome. Therefore, AFP CAR-T cells are safer to use. Moreover, studies have shown that local injection of AFP CAR-T cells into tumors produce deeper, faster, and more lasting anti-tumor responses. Thus, the appropriate route of administration is crucial for optimizing the efficacy of AFP CAR-T cells (102).

## 4 Effects of various components in the immune microenvironment on CAR-T

### 4.1 Effect of treg cells on CAR-T cell therapy for hepatocellular carcinoma

However, CAR-T cell therapy for hepatocellular carcinoma still has significant limitations. When using CAR-T cell therapy, serious adverse reactions may occur, leading to poor treatment effectiveness. In addition, CAR-T cell therapy for hepatocellular carcinoma is still in its early stages, and research in this field is still insufficient. Therefore, it is necessary to be very cautious when using CAR-T cells to treat hepatocellular carcinoma (78). In order to reduce adverse reactions and improve efficacy, researchers have found that using Treg help improve CAR-T treatment for hepatocellular carcinoma.

#### 4.1.1 Umbilical cord blood treg cells eliminate inflammatory responses

During the CAR-T treatment of hepatocellular carcinoma, rapid immune activation reactions are induced, leading to cytokine release syndrome (CRS). CRS causes severe therapeutic toxicity in the body, causing fever, hypoxia, and hypotension, and in severe cases, it is life-threatening (103). Tregs play a good role in eliminating excessive inflammation, maintaining self-immune tolerance, and immune homeostasis (104). The therapeutic effect of Treg cells in metabolic diseases has been proven, and they maintain metabolic balance and prevent inflammation well (105). So, research has found that Treg cells derived from allogeneic umbilical cord blood (UCB) effectively eliminate the inflammatory response caused by CAR-T cells in the treatment of hepatocellular carcinoma. UCB Treg cells may preferentially accumulate at inflammatory sites and interact with antigen-presenting cells, rapidly reducing inflammation. However, it should be noted that UCB Treg cell adjuvant therapy should be administered 3 days after CAR-T cell infusion to minimize the interference of CAR-T cells in the treatment of hepatocellular carcinoma (106).

#### 4.1.2 Immunosuppression is reduced using the C-C Motif chemokine ligand 1- C-C Motif chemokine receptor 8 axis and DNR

**Figure 3:**
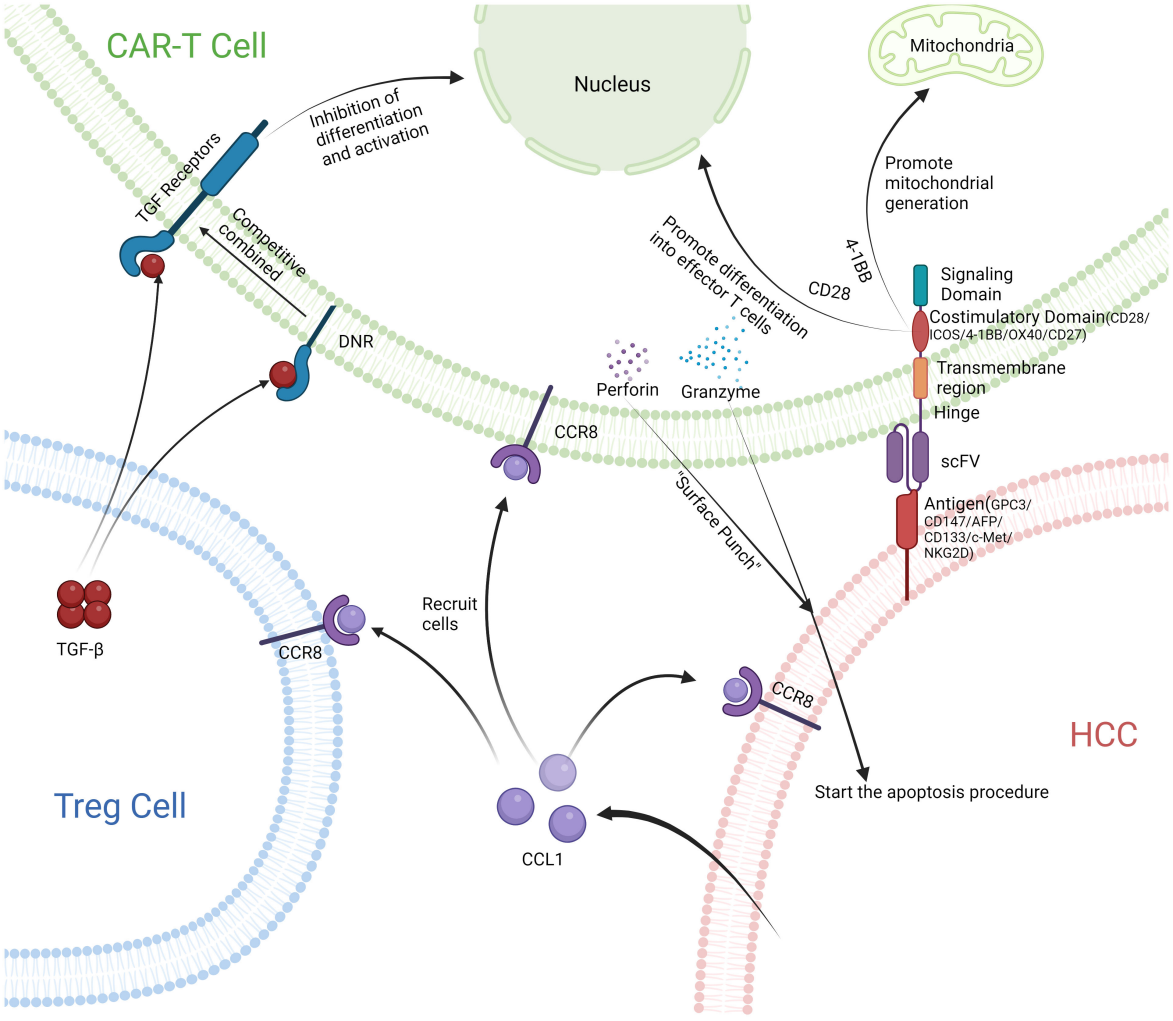
After releasing CCL1 from HCC, Treg cells and CAR-T cells with CCR8 receptor are recruited together. CAR-T cells combine with HCC and release perforin and granzyme to initiate apoptosis of HCC. DNR receptors on the surface of CAR-T cells compete well for binding to TGF-β, Prevent immunosuppression.

Although the inflammatory response caused by CAR-T cells in the treatment of hepatocellular carcinoma has been resolved, excessive immune suppression of Treg cells leading to the promotion of tumor proliferation and metastasis remains a major issue. Treg cells exhibit two primary immunosuppressive mechanisms: one involves the release of transforming growth factor-beta (TGF-β) to perform immune suppression. The second mechanism is achieved when the ligand CCL1 binds to its receptor, C-C chemokine receptor 8 (CCR8) (107). CCR8 is highly expressed in tumors and tumor infiltrating Treg cells. CCR8 is the only receptor of CCL1, so CCL1 activation of the CCR8 receptor on tumor cells promotes their proliferation and metastasis. CCL1 also recruits a large number of Treg cells to the tumor site for immune suppression, promoting tumor growth (108, 109). Utilizing the CCL1-CCR8 axis, which recruits Treg cells to tumor sites, researchers have developed CCR8-CAR-T cells. These CCR8-CAR-T cells are recruited to tumor sites via the CCL1-CCR8 axis, potentially enhancing the efficacy of CAR-T cell therapy. Although CAR-T cells reach tumor cells through this axis, the immunosuppressive effect produced by a large number of Treg cells at the site of tumor infiltration still reduces the therapeutic effect of CAR-T. For this purpose, researchers constructed new CAR-T cells, namely CCR8-DNR-CAR-T cells, to resist the immune suppression produced by Treg. DNR, a truncated form of TGF-β receptor 2, lacks the intracellular signaling components necessary for TGF-β signal transduction. It competes with TGF-β receptor 2 to bind TGF-β, allowing DNR-transduced T cells to clear TGF-β and reduce immunosuppressive reactions (110, 111). Studies have shown that CCR8-DNR-CAR-T cells are recruited in large quantities to tumor sites, but not to other normal organ tissues. CCR8-DNR-CAR-T cells have a long survival and good proliferation ability at the tumor site, and the therapeutic effect on the tumor has also been significantly improved (109) (**Figure 3**).

#### 4.1.3 Lck-inactivated 4–1BB CAR-T resists immunosuppression

Additionally, modifying CAR-T cells to evade Treg cell inhibition is another effective strategy to enhance tumor treatment efficacy. T cells secrete interleukin-2 (IL-2) when stimulated by antigens, and IL-2 promotes the production of Tregs. It has been found that ordinary CAR-T cells promote the production of Treg cells with the help of the IL-2 axis through the co-stimulatory signal CD28 (112). In order to eliminate IL-2 secretion, the researchers replaced two amino acids in the CAR transgene to prevent lymphocyte-specific tyrosine kinase (Lck) from attaching to the CD28 cytoplasmic tail (113), which eliminated IL-2 secretion but also impaired the role of CAR-T cells *in vivo*. However, 4–1BB is a costimulatory glycoprotein on activated immune cells, including lytic and helper T lymphocytes, with its ligand 4–1BBL primarily on antigen-presenting cells like DCs, macrophages, and B cells (114). This interaction enhances T cell proliferation, differentiation, and effector function (115, 116). Cross-linking of 4–1BB in T cells promotes proliferation, memory cell formation, and increases viability and cytokine production, such as IFN-γ and IL-2 (117). In addition, 4–1BB converts T cells with reprogramming tolerance potential into effector T cells with antitumor activity (118), so the introduction of a 4–1BB signaling domain within CAR-T cells made up for this deficiency. 4–1BB CAR-T cells that inhibit Lck activation are not only active at tumor sites but also resist Treg-mediated immunosuppression. Studies have shown that 4–1BB is targeted by Treg cells in various cancers and plays a crucial role in regulating effector T cell responses, demonstrating significant therapeutic effects within the immune microenvironment (119, 120).

#### 4.1.4 Optimization of CAR-T cells

Although improvements have been made in the effects and adverse reactions of CAR-T cells, significant challenges remain in developing standardized, safe, and reliable CAR-T cells (121). To address this, the construction process has been optimized to enhance the efficacy of CAR-T cells against tumors. For instance, incorporating an apoptosis switch in CAR-T cells that activates caspase9 (iCasp9) is beneficial. Activation of iCasp9 by a synthetic dimerizer induces apoptosis in CAR-T cells, allowing for controlled regulation of their activity based on specific situations. This improves the safety of CAR-T cell therapy. Additionally, gene editing is employed to develop multifunctional CAR-T cells. In hepatocellular carcinoma, PD-L1 is upregulated and expressed by various activated immune cells including macrophages, B cells, dendritic cells, and T cells (122). PD-1 is expressed on immature thymocytes, activated CD4 and CD8+ T cells, B cells, dendritic cells, and natural killer (NK) cells. PD-L1, when expressed on hepatocellular carcinoma cells, bind to PD-1 on T cells, inducing tolerance by inhibiting their ability to mount an effective immune response against specific cancer antigens (123). Regulatory T cells express PD-L1, which bind to inhibitory receptors on NK cells, thereby suppressing their cytotoxic function and preventing the direct killing of tumor cells (124). The interaction of PD-L1 with PD-1 on immune cells transmits an inhibitory signal that decreases the cytotoxic activity of effector T cells, aiding the survival of tumor cells (125). Activated PD-1/PD-L1 complexes modulate T cell receptor and CD28 co-stimulatory signaling pathways by recruiting SHP1/2, which dephosphorylates these receptors, leading to reduced T cell activation and increased apoptosis, promoting immune escape (126). SHP1/2 remove phosphate groups, inhibit T cell activation, increase cytokine production, promote expression of apoptotic molecules, ultimately leading to T cell apoptosis and non-responsiveness, driving tumor immune escape and progression (127). Both PD-1 and CD276 are members of the B7 family, making their effects on the tumor microenvironment similar. The binding of CD276/PD-1 and the corresponding receptor exert a synergistic effect, thereby jointly inhibiting the proliferation of T cells and the secretion of related cytokines (128, 129). Therefore, by utilizing gene editing technology to disrupt genes like PD-1 and CD276 in CAR-T cells, researchers inhibit the tumor microenvironment and enhance the therapeutic efficacy of CAR-T cells (130).

#### 4.1.5 Regulatory T cells depletion enhances CAR-T efficacy

For Treg cells, researchers have found that the tumor microenvironment significantly induces high expression of histone demethylase JMJD1C in tumor Tregs. Inhibiting JMJD1C expression does not affect the development and function of peripheral Tregs but reduces tumor-associated Tregs, consequently slowing tumor growth. This effect is primarily due to JMJD1C’s role in promoting PD-1 expression and inhibiting AKT signaling and IFN-g production in tumor-associated Tregs. The use of JMJD1C inhibitors significantly inhibits tumor growth and has good application prospects (131).

### 4.2 Tumor-associated macrophages influence CAR-T therapy in the treatment of HCC

Tumor-associated macrophages (TAMs) inhibit the anti-tumor immune response within the tumor microenvironment, thereby promoting tumor growth through various mechanisms and diminishing the efficacy of CAR-T therapy (132). First, TAMs express the inhibitory molecule PD-L1, which binds to PD-1 on CAR-T cells, reducing their cytotoxicity and efficacy. Secondly, TAMs secrete several immunosuppressive cytokines such as IL-10 and TGF-β. IL-10 weakens the response of CD8^+^ T cells (133), while TGF-β excludes T cells, including CAR-T cells, from the tumor, significantly impairing their function. Thirdly, TAMs inhibit the recruitment of T cells to tumor sites by producing peroxynitrites and nitrifying CCL2 and CCL5, thereby preventing chemotaxis of CAR-T and other T cells to the tumor, reducing their effectiveness (134). Finally, TAMs produce VEGF and other growth factors that support blood vessel and tumor survival.

### 4.3 Effect of cancer-associated fibroblasts on CAR-T therapy in HCC

Cancer-associated fibroblasts (CAFs) inhibit the infiltration and function of T cells, including CAR-T cells, within the immune microenvironment, thereby reducing the efficacy of CAR-T therapy. As a significant component of the tumor microenvironment, CAFs promote the production of TAMs, which in turn inhibit CAR-T cell function (135). Additionally, CAFs secrete immunosuppressive cytokines like IL-10 and TGF-β, further suppressing T cell activity. CAFs also enhance the recruitment and differentiation of Tregs by producing chemokines (136), indirectly inhibiting CAR-T cell function. Moreover, CAFs interfere with T cell-dependent immune responses by regulating myeloid-derived suppressor cells (MDSCs). By secreting CXCL12/SDF1, CAFs recruit monocytes to the tumor site, which are then induced to differentiate into MDSCs through IL-6-mediated STAT3 activation, thus altering CAR-T cell proliferation and function and diminishing their therapeutic effect (137).

### 4.4 Effects of metabolites on CAR-T therapy

The unique metabolic profile of tumor cells creates a nutrient-restricted, hypoxic, and lactate-rich environment in the tumor microenvironment. Hypoxia is closely associated with cancer cell resistance to radiotherapy and chemotherapy, as well as impaired immune cell function, thus impacting the efficacy of CAR-T therapy. Researchers observed that hypoxia inhibits CAR-T cell proliferation and differentiation and reduces granzyme B and cytokine production without affecting their frequency and cytotoxicity (138). To enable CAR-T cells to function in hypoxic conditions, researchers integrated hypoxia-induced transcription factor-1α (HIF-1α) hypoxia response element (HRE) into CAR-T cells, creating a hypoxia-induced transcription amplification system that significantly enhances anti-tumor efficacy under hypoxia (139).

Tumor proliferation also results in increased glucose consumption and lactate production, causing metabolic acidosis and a low pH state in the tumor microenvironment. Acidosis inhibits glycolysis and oxidative phosphorylation, affecting CAR-T cell metabolism. Low pH induces V-domain Ig suppressor of T cell activation (VISTA), which inhibits T cell activation, proliferation, and cytokine production, thus affecting CAR-T therapy (140). Researchers found that pretreating T cells with lactate *in vitro* enhance CAR-T therapy by improving their stemness and anti-tumor immunity (141).

### 4.5 Impact of PD-1/PD-L1 on CAR-T therapy

PD-1/PD-L1 interactions assist tumor cells in immune evasion and impact CAR-T therapy by reducing T cell glycolysis, impairing T cell effector functions, and limiting glucose availability for T cells (142). Initially, researchers used monoclonal antibodies against PD-1 to block the PD-1/PD-L1 axis, but this approach had serious adverse reactions and increased the risk of immune tolerance breakdown. To enhance CAR-T therapy for solid tumors like HCC, researchers combined CAR-T cells with PD-1 knockout (KO) therapy to improve anti-tumor activity and safety (143).

### 4.6 Effect of exosomes on CAR-T therapy

Exosomes are extracellular vesicles that carry bioactive molecules and influence recipient cells’ pathophysiological processes. Tumor cells release small extracellular vesicles (sEVs) after CAR-T therapy, promoting PD-L1 expression and inhibiting CAR-T cell efficacy. Exosome inhibitors, such as GW4869 and Nexinhib20, have been shown to enhance CAR-T therapy by improving CD8^+^ T cell infiltration and activation (144).

### 4.7 Effect of extracellular matrix on CAR-T therapy

In HCC, excessive collagen and hyaluronic acid in the ECM lead to matrix hardening, poor diffusion, decreased oxygen content, and delayed material exchange in the tumor microenvironment(TME) (145).ECM stiffness suppresses T lymphocyte-mediated anti-tumor responses and reduces CAR-T cell infiltration (146). Researchers have incorporated strategies to inhibit hyaluronan synthesis and enhance degradation into CAR-T therapy to decompose ECM and promote T cell penetration (147). Additionally, combining CAR-T therapy with tumor vaccines targeting ECM components softens the ECM and improve T cell infiltration (148).

## 5 Challenges and future outlook

Although CAR-T therapy is effective in treating liver cancer cells, there are still many deficiencies and challenges. Firstly, various substances in the immune microenvironment weaken the efficacy of CAR-T in hepatocellular carcinoma. Among these, several obstacles affecting the function of CAR-T in the tumor microenvironment (TME) include hypoxia, high lactate levels, activation of the PD-1/PD-L1 axis, and accumulation of the extracellular matrix (ECM) (138, 140, 142, 145).Secondly, CAR-T therapy also faces limitations such as long treatment cycles, high costs, and the need for standardization, safety, and reliability (130). Additionally, CAR-T therapy has certain hepatotoxicity, which may damage the normal function and proliferation of hepatocytes. Currently, CAR-T cells are still insufficiently infiltrated in the environment of hepatocellular carcinoma, so CAR-T cell therapy still needs improvement.

However, with technological advancements, these issues are gradually being addressed. For example, anti-hypoxia CAR-T cells have been developed by introducing hypoxia-inducible factor elements (139), and immune checkpoint molecules such as PD-1/PD-L1 have been knocked out using CRISPR/Cas9 gene editing technology (143). Combining CAR-T cell therapy with anti-PD-1/PD-L1 monoclonal antibodies to enhance the anti-tumor response is a promising research direction. Additionally, combined therapy is used to integrate anticancer drugs with CAR-T therapy to improve the efficacy of CAR-T cells by enhancing the tumor microenvironment. Finally, with future research directions and the use of technological advancements, the field of CAR-T treatment for liver cancer progresses towards more effective and lasting treatments, ultimately improving patient prognosis.

## 6 Summary

This review has highlighted the significant role of various components of the tumor microenvironment in aiding immune escape in hepatocellular carcinoma, particularly emphasizing the importance of regulating T cells in the growth and spread of cancer cells. To counteract the immune escape mechanisms of hepatocellular carcinoma and enhance T cell therapy efficacy, researchers have explored the potential of CAR-T cell therapy. In conclusion, a thorough understanding of how T cells regulate immune escape in hepatocellular carcinoma offers valuable insights for theoretical and clinical advancements in developing more effective immunotherapies for this cancer. We hope that our research will lead to more treatment options and improve the quality of life for patients with hepatocellular carcinoma.

## Author contributions

GD: Writing – original draft. CD: Writing – original draft. PS: Writing – review & editing. SW: Writing – review & editing, Funding acquisition. JL: Writing – review & editing. LM: Writing – review & editing, Funding acquisition.
