# Supplementary material for: Regulatory T cells and immune escape in HCC: understanding the tumor microenvironment and advancing CAR-T cell therapy
Source: Front Immunol. 2024 Jul 29;15:1431211. doi: 10.3389/fimmu.2024.1431211 (PMC11317284; doi:10.3389/fimmu.2024.1431211)
Supplement: Supplementary file 1 [file DataSheet_1.pdf]

Components		Functions
Immune cells	T cells	CTLs directly kill tumors by secreting granzymes and per forins, while Tregs promote t umor cell escape directly by secreting cytokines (such as TGF-β and IL-10) and by in direct pathways (such as CA F activity)
	Natural killer cells (NK cells)	Inhibit tumor progression thr ough perforin and granzyme B
	B cells	Secrete antibodies and cytoki nes, participate in tumor im mune response, and promote the differentiation of Treg
	Dendritic cells (DCs)	Modulation of immune respo nses through antigen presenta tion and cytokine secretion
	Neutrophils	Secrete IL-10 and CCL2 to promote tumor cell growth, metastasis and angiogenesis
	Macrophages	Regulate T cells by releasing cytokines (such as TGF-β, I L-10) and chemokines (such as CCL22), thereby indirectl y promoting tumor cell esca pe
Stromal cells	Hepatic stellate cells (HSCs)	After activation, it secretes a ngiogenic growth factors, pro moting the formation of new blood vessels and supportin g tumor growth
Tumor cells	Cancer-associated fibroblasts (CAFs)	Promotes tumor cell invasion and metastasis by secreting collagen and extracellular ma trix proteins
Enzymes	Glucose-6-phosphate dehydrogenase (G6PD)	Boosts metabolic pathways s uch as the pentose phosphate pathway, supporting tumor cell growth and DNA synthe sis
	Matrix metalloproteinases (MMPs)	Degrade extracellular matrix

		and promote tumor metastasis
	CD39,CD73	The synergistic effect of the two inhibits the function of effector T cells
Cellular factors	Vascular endothelial growth factor (VEGF)	Stimulates new blood vessel formation, inhibits the antigen presentation function of dendritic cells, and enhances the immunosuppressive function of Treg
	Transforming growth factor-β (TGF-β)	Induces epithelial-mesenchymal transition (EMT) of liver cancer cells, enhances migration and invasion ability, and promotes immune escape
	Interferon-γ (IFN-γ)	Synergizes with IL-1β to enhance PD-L1 expression and reduce Treg suppressive activity
	Interleukin 10 and Interleukin 35 (IL-10 and IL-35)	Inhibits immune response by inhibiting cell proliferation, reducing the production of inflammatory mediators, promoting the development and function of Treg cells, and assisting immune escape
Immune-related proteins	Leukocyte function associated antigen-1 (LFA-1)	Forms aggregates with dendritic cells, inhibiting the co-stimulation process between dendritic cells and effector T cells
Ligands and receptors	Programmed Death-1/Programmed Death - Ligand 1 (PD-1/PD-L1)	After binding, it transmits inhibitory signals, reduces T cell activity, proliferation and cytokine production, and helps tumor immune escape
	CD276	Inhibit T cell activity, reduce cytokine secretion, and stimulate angiogenesis
	CD40	Stimulates the production of cytokines and chemokines, thereby promoting anti-tumor effects

	V-domain Ig suppressor of T-cell activation (VISTA)	Inhibit T cell proliferation and activation and Treg transformation, and reduce the production of cytokines
	4-1BB	Interaction with 4-1BBL enhances T cell proliferation, differentiation and effector function
Chemokines	C-C Motif Chemokine Ligand 1 (CCL1)/ Characteristic Chemokine Receptor 8 (CCR 8)	Enhance the immunosuppress ive activity of Treg and pro mote tumor cell proliferation, anti-apoptosis and inflamma tory response
	C-C Motif Chemokine Ligand 22 (CCL22)/Characteristic Chemokine Receptor 4 (CCR4)	Recruited Tregs gather at tu mor sites and induce local i mmune escape in hepatocellu lar carcinoma by secreting a large amount of IL-10 and T GF-β while inhibiting IL-2 s ecretion
Metabolites	lactic acid	Transform the tumor microen vironment into an environme nt suitable for tumor cell pr oliferation
Exosomes		Promotes angiogenesis and t umor growth, regulates immu ne escape, and affects the tu mor microenvironment
Extracellular matrix		Helps tumor cells invade an d metastasize, and promotes the metastasis of hepatocellul ar carcinoma
